# Clinical and Microbiological Profile of HIV/AIDS Cases with Diarrhea in North India

**DOI:** 10.1155/2012/971958

**Published:** 2012-12-27

**Authors:** Arun Kumar Jha, Beena Uppal, Sanjim Chadha, Preena Bhalla, Roumi Ghosh, Prabhav Aggarwal, Richa Dewan

**Affiliations:** ^1^Department of Microbiology, Maulana Azad Medical College, University of Delhi, Bahadur Shah Zafar Marg, New Delhi 110002, India; ^2^Department of Medicine, Maulana Azad Medical College, University of Delhi, Bahadur Shah Zafar Marg, New Delhi 110002, India

## Abstract

Intestinal infections are a significant cause of morbidity and mortality in people living with HIV/AIDS (PLWHA) especially in developing countries. The present study was conducted to assess the clinical and microbiological spectrum in HIV/AIDS cases with diarrhea and to correlate the occurrence of such pathogens with stool characters, HIV seropositivity status, and CD4 counts. Stools from 154 HIV seropositive subjects and 50 HIV negative controls were examined by direct microscopy, fecal cultures, and serological tests (*Clostridium difficile *Toxin A, *Cryptosporidium *antigen, and *Entamoeba histolytica *antigen ELISA). CD4 T cell enumeration was done using FACS count (Becton Dickinson). The study showed a male preponderance (112 males and 42 females). Weakness, abdominal pain, and anorexia were the most common symptoms. Coccidian parasites were the most common cause of diarrhea in HIV seropositive cases. *C. parvum *was seen in 60.42% while *Isospora belli *in 9.03%. Amongst the bacterial pathogens *C. difficile *was detected in 18.06%, diarrheagenic *Escherichia coli *in 11.11%, and *Shigella *spp. in 2.78%. Pathogen isolation rates were more in HIV seropositive cases and subjects with low CD4 T lymphocyte counts. Regular monitoring of CD4 T lymphocyte counts and screening for enteric pathogens will help improve the quality of life for PLWHA.

## 1. Introduction

Infection with human immunodeficiency virus (HIV) imposes monumental suffering on afflicted individuals, and in the developing world in particular it places a great burden on the medical system [1]. For a poverty-stricken and disease-ravaged country like India, the scourge of HIV/AIDS is most unfortunate. One of the major health problems among HIV seropositive patients due to a waning immunity is superimposed opportunistic infections, and it is often seen that during the course of the disease patients become a microbial zoo [2, 3]. Patients may have several such concurrent infections, resulting in clinical conditions that pose diagnostic and therapeutic challenges [4–6].

Diarrhea is one such very common clinical condition in HIV/AIDS and has been included as a criterion for defining a case of AIDS [7, 8]. Episodes of diarrhea may be acute and brief, intermittent or recurrent, or, in some cases, chronic and severe. Diarrhea may significantly diminish patients' quality of life and if it persists may cause dehydration, poor nutrition, and weight loss [9, 10]. Diarrhea has been associated with 50% of HIV/AIDS patients in the developed world and in up to 100% of patients residing in developing countries [11–13].

The causes of diarrhea in AIDS can be infectious or noninfectious. Noninfectious diarrhea could be due to ART-related adverse effects and HIV enteropathy [14]. Several studies have shown that infectious diarrhea in HIV/AIDS is caused by a variety of pathogens including parasites, bacteria, viruses, and fungi. There is no specific combination of intestinal pathogens in HIV-associated diarrhea, and the etiological agents vary from patient to patient and from country to country depending on the geographical distribution, endemicity, seasonal variation of the enteric pathogens, and also on the immune status of the patient [15–18]. A diagnostic workup including direct microscopy, fecal cultures, and serological tests to detect specific antigen and/or specific antibody is needed for each patient as most of these infectious agents are treatable.

With the previous background in mind, this study was undertaken to assess the clinical and microbiological spectrum in HIV/AIDS cases with diarrhea registered in Delhi's largest tertiary care hospital catering to patients not only from Delhi but also from the neighboring states of Uttar Pradesh, Haryana, Punjab, and Himachal Pradesh. The motive was to understand the microbial etiologies of diarrhea in such cases so that appropriate medical investigations, specific therapy, and adequate nutritional counseling can help reduce the socioeconomic and medical costs for this disease in our country.

## 2. Materials and Methods

### 2.1. Study Population

One hundred and fifty-four HIV seropositive adult subjects with diarrhea attending the ART clinic of Lok Nayak Hospital affiliated to Maulana Azad Medical College, New Delhi, India, were recruited for this study irrespective of their ART status. Only those HIV seropositive subjects with diarrhea were enrolled who had not received any specific antidiarrheal therapy in the last two weeks. Fifty age- and sex-matched randomly selected adult HIV seronegative subjects with symptoms of diarrhea were also enrolled as control group who come for routine examinations of their stool samples to the microbiology laboratory of Maulana Azad Medical College, New Delhi. Subjects who had received any specific antidiarrheal therapy in the last two weeks and/or were not sure of their HIV status were excluded from the control group.

### 2.2. Study Design

This study was conducted from April 2008 to June 2011. This was a cross-sectional analysis to determine the clinical and microbiological profile of diarrhea in AIDS cases and HIV seronegative control subjects. At enrollment informed consent was obtained and each study participant was asked to complete a questionnaire which consisted of sociodemographic and personal details, history of diarrheal episodes, clinical signs and symptoms, and so forth. Fecal specimens were requested from all the participants. Samples were collected in a clean wide mouth screw capped disposable plastic container and transported to the microbiology laboratory by the patients themselves on the same day avoiding any unnecessary delay.

### 2.3. Definition of Diarrhea

Diarrhea was defined as the passage of three or more loose or watery bowel movements in a 24-hour period. Acute diarrhea was defined as diarrhea which lasted 7 days or less at the time of presentation.Persistent diarrhea was defined as diarrhea which lasted for more than 7 days but less than 14 days at presentation. Diarrhea was called chronic if it lasted for more than 14 days [19].

### 2.4. Laboratory Examination

All fecal specimens were subjected to a battery of microbiological examinations. The specimens were stored at 4 degree Celsius if there was a delay in processing. The color, consistency, and presence of blood/mucus/worms in the stool specimens were recorded. A loopful of sample was emulsified in a drop of saline and Lugol's iodine on a slide and examined under the microscope for the presence of trophozoites of *Entamoeba histolytica*, *Giardia lamblia*, RBCs, pus cells, helminthic ova, and cyst. Stool smears were prepared, heat fixed, and stained by the Gram's, Kinyoun's (modified acid fast stain), and trichrome stain. All samples were cultured directly as well as after enrichment in Selenite F Broth and Alkaline Peptone Water onto Xylose Lysine Deoxycholate agar and Bile Salt Agar respectively. Specialized selective media, charcoal cefoperazone deoxycholate agar (CCDA) was used for isolation of *Campylobacter jejuni* which was incubated in a microaerophilic environment at 42°C for 48 hrs. For isolation of *Aeromonas *spp. and *Yersinia enterocolitica*, stool samples were cultured on Aeromonas selective media and Yersinia selective media, respectively. The organisms were identified on the basis of their colony characteristics, biochemical tests, and serologically by slide agglutination test using commercially available specific antisera. Detection of *C. difficile* Toxin A, *Cryptosporidium* antigen, and *E. histolytica* antigen in stool samples was done by commercially available Enzyme Immunoassay kits.

The CD4 T lymphocyte count of all participants was determined by the FACS count by Becton Dickinson.

### 2.5. Statistical Analysis

To study the correlation between the frequencies of enteric pathogens and stool consistency the Kruskal-Wallis test was applied. To study the relation between the isolation rate of intestinal pathogens and the HIV seropositivity status, and CD4 T lymphocyte counts, the chi-square test and the Fisher's exact test were used.

## 3. Results

Out of the one hundred and fifty-four HIV seropositive subjects recruited 112 (72.73%) were males and 42 (27.27%) were females. 64.94% of the study subjects were in the age group 26–35 years, the sexually active age group. The mean age of the participants in our study was 32.36 years, and the age ranged between 18 and 68 years. Majorities (23%) of our subjects were illiterate, and 21.7% had received education up to the primary school level. 

Table 1 shows the distribution of cases as per the clinical symptoms recorded at the time of recruitment of cases. Weakness, abdominal pain, and anorexia were the most common symptoms associated with diarrhea in HIV seropositive cases while abdominal pain, vomiting, and fever were the most common complaints in the HIV seronegative control group.

Out of 154 HIV seropositive cases enrolled, only 144 participants submitted their fecal specimens. Majority (60.39%) of the HIV seropositive cases had chronic diarrhea (Table 2) while most (72%) of the HIV seronegative subjects had acute diarrhea.

Table 3 shows the enteropathogens isolated in relation to stool consistency. Certain pathogens (*C. parvum* and *I. belli*) were more frequently detected in watery stools, and this was found to be statistically significant (*P* value < 0.05), whereas the bacterial pathogens (*C. difficile*, diarrheagenic *E. coli,* and *Shigella *spp.) were found to be significantly more frequent in the formed stools (*P* value < 0.05). Coccidian parasites were the most common cause of diarrhea in HIV seropositive cases, *C. parvum* being detected in 60.42% cases. Amongst the bacterial pathogens *C. difficile* topped the list with 18.06% cases showing *C. difficile* positivity. *Candida albicans* was isolated in 25.69% of our cases. 

Table 4 shows the frequency of intestinal pathogens in relation to the HIV status of the study cases. *C. parvum*, *C. difficile,* and *C. albicans* were found to be significantly more common in the HIV-seropositive study cases than the HIV-negative control group (*P* value < 0.05). 

Overall the rate of isolation of pathogens causing diarrhea in HIV was higher in HIV seropositive individuals with CD4 counts less than 200 cells/*μ*L as compared to HIV seropositive individuals with CD4 counts more than 200 cells/*μ*L. However, the isolation rate was significantly higher for *C. parvum*, *I. belli*, *C. difficile,* and *C. albicans* only (*P* value < 0.05) in patients with CD4 < 200 cells/*μ*L as shown in Table 5.

## 4. Discussion

Diarrhea is the second leading cause of hospital visits in the developing nations in patients with HIV/AIDS [20]. The etiology of diarrhea in AIDS is multifactorial. Expectedly infectious etiologies lead the list in developing nations in contrast to noninfectious etiologies in developed nations. There are many studies on the etiological agents of diarrhea in HIV/AIDS from various parts of North India [21–23]. But there are very few reports on the diarrheal pathogens isolated in HIV/AIDS cases in relation to CD4 T lymphocyte counts and stool characteristics from New Delhi. Our study divulges the infectious etiological agents of diarrhea in HIV/AIDS patients from New Delhi's busiest and largest tertiary care hospital located at the heart of the city. This study also looks into the correlation of diarrheal agents isolated with HIV seropositivity status, stool characteristics, and CD4 T lymphocyte counts.

 The present study shows a preponderance of male cases (112 males out of 154 cases) with a male-to-female ratio of 2.66 : 1 as shown by other studies conducted on HIV-positive cases with diarrhea in India [24]. Predominance of male cases may be due to their migration to the metropolitan cities (Delhi being one of such type) in search of work. Staying away from their spouse for longer periods and males being promiscuous by habit resulted in acquiring HIV infection. Moreover, the male preponderance might have been due to the fact that in the existing social milieu in India, females do not seek medical care fearing ostracism and loss of family support. The mean age of the participants in our study was 32.36 years with the most common age group being 26–35 years. This section of the population is more affected because they are sexually more active. Similar results were obtained in a study from South India where median age of HIV seropositives with diarrhea was 34 and the mean age was 36 years [25]. 

The most frequent clinical findings in the HIV seropositive cases with diarrhea in our study were weakness (64.93%), abdominal pain (61.69%), and anorexia (51.95%). This is similar to what has been reported by Chhin et al. from Cambodia where abdominal pain (90.3%), fever (86.7%), and weakness (80%) were the most common presenting complaints in HIV-positive cases with diarrhea [26]. Weakness and anorexia were the symptoms found to be significantly (*P* value < 0.05) associated with diarrhea in HIV-positive cases as compared to the HIV seronegative control group (Table 1). Chronic diarrhea was significantly more (60.39%; *P* value < 0.05) in the HIV positives than the HIV negatives (Table 2). Another study from North India has reported chronic diarrhea in 69.3% HIV cases [24]. Vomiting, fever, and nausea were more frequently seen in the HIV seronegative group with diarrhea (association found to be statistically significant; *P* value < 0.05), and most of the subjects in this group had acute diarrhea (36/50; *P* value < 0.05). This may be due to the fact that diarrhea in HIV seronegatives is most probably due to acute infectious causes which is more likely to be associated with fever, nausea, and vomiting. And diarrhea which is chronic in nature, as is seen in the HIV-positive cases, is more likely to be associated with anorexia, weakness, and weight loss.

In our study *C. parvum* and *I. belli* were significantly more commonly seen to be associated with watery stools while the bacterial enteropathogens (*C. difficile*, diarrheagenic *E. coli,* and *Shigella *spp.) were more common in the formed stools (*P* value < 0.05). A study from Zambia reports that infections with *C. parvum*, *I. belli*, and *G. lamblia *are associated with increasing water content of the stool samples in HIV-positive cases with diarrhea [27]. Higher positivity associated with watery stools could be due to infection with the more invasive and virulent enteropathogens causing more inflammation leading to watery diarrhea and increased shedding. This observation of the consistency of the stools of HIV/AIDS patients could help in the presumptive diagnosis of the intestinal etiological agents and allow parasitological investigation to be targeted at the cases most likely to be found positive.

We found that intestinal parasites are the most common enteric pathogens associated with diarrhea in the North Indian HIV-positive population. A study from Chennai also documents the prevalence of enteric parasites in HIV patients with diarrhea [28]. The microbiological profile seen in our HIV seropositive cases is typical of what has been reported by other Indian studies [29], intestinal coccidian parasites being the most common enteric pathogens isolated amongst which *C. parvum* was the most frequent (60.42% of the cases).* Cryptosporidium* positivity rate in HIV-positive patients with diarrhea has been reported to be in the range of 5.71%–22.8% by various Indian authors [20–23]. The substantially high *Cryptosporidium* positivity in our study could be due to the use of more than one method of detection of *Cryptosporidium,* for example, Modified Kinyoun's method and ELISA. It could also be due to the fact that majority of our cases had watery and semiformed stools. Geographical and seasonal variations have also been seen to impact the prevalence of this parasite. *Cryptosporidium* causes profuse and watery diarrhea in AIDS patients and may produce fluid loss of up to 10 liters per day. Small environmentally resistant oocysts, low infective dose (10–100 oocysts), and oocysts being resistant to disinfectants are some of the factors which impact the epidemiology of *Cryptosporidium* infection. As no effective cure is available for cryptosporidiosis especially in immunocompromised individuals, it usually has a poor prognosis [2]. The isolation rate of *Cyclospora* (1.39%) and *Microsporidia* (0.69%) was quite low in the present study. The reason for this low detection may be that these pathogens are shed intermittently and may not have been present in the fecal samples submitted or may have been probably overlooked by the inexperienced microscopist. Repeated examination of fecal samples is recommended for their detection. The diagnostic methods like transmission electron microscopy, histochemistry, immunofluorescent antibody staining, and PCR-based methods should improve and simplify their detection in clinical samples [30, 31]. Some authors have reported a low percentage of *Microsporidium *(1.69%) and *Cyclospora *(1.69%) while others have reported a high percentage (41% and 2.6%) from India [28, 32]. Ascaris lumbricoides was seen in 5.56% of our cases probably due to expulsion in the feces with repeated flushing of intestinal contents in diarrhea. *G. lamblia* and *E. histolytica* were found in 3.7% and 7.41% of the cases. The presence of these parasites reflects poor environmental hygiene and sanitation. They have been seen to cause chronic diarrhea in immunocompromised hosts.

In our study *C. difficile* was the most common bacterial pathogen identified (18.06%). This points out that *C.difficile* is a common enteric pathogen responsible for diarrhea in HIV-infected patients since they are subjected to repeated antibiotic therapy due to opportunistic infections, and clinicians should keep this pathogen as a differential diagnosis when searching for the cause of diarrhea in HIV/AIDS especially in developing countries like ours where antibiotic access is unregulated. A study from Nigeria has reported the prevalence of *C. difficile* infection to be 43% and 14% for HIV-positive inpatients and outpatients, respectively [33]. *C. albicans* was seen in 25.69% cases in our study. This may be due to low immunity and frequent use of antibiotics. A high rate of isolation of *C. albicans* (36%) in HIV-positive cases with diarrhea has been reported in a previous study from our department in past [34].

*C. parvum*, *C. difficile,* and *C. albicans* were significantly more common in the HIV-positive group than the HIV-negative control group (*P* value < 0.05). This suggests that immunodeficient state in AIDS makes PLWHA more susceptible to such infections, and once established they are not able to prevent the proliferation or clear the infecting agent. This finding corresponds well with other studies [8, 27] and is a common observation in HIV/AIDS.

In our study HIV seropositive cases with CD4 counts <200 cells/*μ*L had a higher rate of infection with certain pathogens, and this was found to be statistically significant as shown in Table 5. The most common amongst these pathogens were the opportunistic gastrointestinal parasites, *C. parvum,* and *I. belli* which cause infection when there is a downregulation of the immune system as is seen with decreasing CD4 levels. The isolation rates decreased with the increase in the CD4 cell counts due to immune reconstitution seen after effective administration of HAART. This is in accordance with the study conducted by Tuli et al. who found an inverse relationship between isolation rates of enteric pathogens and CD4 counts [24]. 

## 5. Conclusions

The present study has revealed that the majority of HIV-positive cases had chronic diarrhea, coccidian parasites were the major pathogens incriminated in causation of this diarrhea, and cases with watery stools had a higher rate of pathogens detected. Isolation rates were also higher in those with CD4 counts <200 cells/*μ*L. 

To sum up our study highlights the importance of early diagnosis of intestinal pathogens causing diarrhea in HIV/AIDS as this would contribute to significantly reducing the morbidity and mortality associated with it. This study also emphasizes the need to establish appropriate diagnostic facilities for identification of enteric organisms in the stool specimens and their ready availability at the peripheral health centers in our country where the HIV/AIDS disease burden is concentrated. This is vital as a delay in transportation of stool specimens to the far-off urban laboratories, and lack of motivation on the part of the unaware patient plays a major role in the case fatalities associated with this disease.

## Figures and Tables

**Table 1:** Clinical symptoms of cases with diarrhea.

Symptoms	HIV seropositive No. (percentage) (*n* = 154)	HIV seronegative No. (percentage) (*n* = 50)	*P* value (chi-square test)
Weakness	100 (64.93%)	15 (30.00%)	0.0001
Abdominal pain	95 (61.69%)	35 (70.00%)	0.372
Anorexia	80 (51.95%)	7 (14.00%)	0.0001
Fever	30 (19.48%)	27 (54.00%)	0.0001
Nausea	25 (16.23%)	24 (48.00%)	0.0001
Vomiting	21 (13.64%)	30 (60.00%)	0.0001
Blood in the stools	10 (6.50%)	5 (10.00%)	0.608

**Table 2:** Duration of diarrhea in study subjects.

Duration	HIV seropositive No. (percentage) (*n* = 154)	HIV seronegative No. (percentage) (*n* = 50)	*P* value (chi-square test)
<1 week (acute diarrhea)	32 (20.78%)	36 (72.00%)	0.0001
1-2 weeks (persistent diarrhea)	29 (18.83%)	8 (16.00%)	0.810
>2weeks (chronic diarrhea)	93 (60.39%)	6 (12.00%)	0.0001

**Table 3:** Frequency of enteric pathogens isolated in relation to stool consistency (*n* = 144).

Organism	Formed (*n* = 30)	Semiformed (*n* = 68)	Loose/watery (*n* = 46)	Total (*n* = 144)	*P* value*
*C. parvum *	11 (36.67%)	39 (57.35%)	37 (80.43%)	87 (60.42%)	0.0001
*I. belli *	1 (3.33%)	4 (5.88%)	8 (17.39%)	13 (9.03%)	0.022
*Cyclospora* spp.	0	0	2 (4.35%)	2 (1.39%)	—
*Microsporidium *spp.	0	0	1 (2.17%)	1 (0.69%)	—
*E. histolytica *	2 (6.67%)	5 (7.35%)	0	7 (4.86%)	0.121
*G. lamblia *	0	3 (4.41%)	0	3 (2.08%)	—
*A. lumbricoides *	3 (10.00%)	1 (1.47%)	1 (2.17%)	5 (3.47%)	0.125
*C. difficile *	15 (50.00%)	9 (13.23%)	2 (4.35%)	26 (18.06%)	0.0001
Diarrheagenic* E. coli *	10 (33.34%)	5 (7.35%)	1 (2.10%)	16 (11.11%)	0.0001
*Shigella *spp.	3 (10.00%)	1 (1.47%)	0	4 (2.78%)	0.018
*C. albicans *	10 (33.34%)	24 (35.29%)	3 (6.52%)	37 (25.69%)	0.002

*P value calculation using Kruskal-Wallis test.

**Table 4:** Frequency of enteric pathogens in relation to the HIV status of study subjects.

Organism	HIV seropositive cases (*n* = 144)	HIV seronegative subjects (*n* = 50)	*P* value (chi-square test)
*C. parvum *	87 (60.42%)	1 (2.00%)	0.0001
*I. belli *	13 (9.03%)	1 (2.00%)	0.082
*Cyclospora *spp.	2 (1.39%)	0	—
*Microsporidium *spp.	1 (0.69%)	0	—
*E. histolytica *	7 (4.86%)	1 (2.00%)	0.343
*G. lamblia *	3 (2.08%)	2 (4.00%)	0.726
*A. lumbricoides *	5 (3.47%)	1 (2.00%)	0.514*
*C. difficile *	26 (18.06%)	3 (6.00%)	0.040
Diarrheagenic* E. coli *	16 (11.11%)	2 (4.00%)	0.166*
*Shigella * spp.	4 (2.78%)	0	—
*C. albicans *	37 (25.69%)	2 (4.00%)	0.0001

*P value calculation using Fisher's exact test.

**Table 5:** Frequency of enteropathogens in relation to CD4 counts in HIV seropositive cases (*n* = 144).

Organism	CD4 < 200 cells/*μ*L (*n* = 54)	CD4 ≥ 200 cells/*μ*L (*n* = 90)	*P* value (chi-square test)
*C. parvum *	47 (87.04%)	40 (44.45%)	0.0001
*I. belli *	11 (20.37%)	2 (2.23%)	0.0001*
*Cyclospora *spp.	2 (3.70%)	0	—
*Microsporidium *spp.	1 (1.85%)	0	—
*E. histolytica *	4 (7.41%)	3 (3.34%)	0.403*
*G. lamblia *	2 (3.70%)	1 (1.11%)	0.556*
*A. lumbricoides *	3 (5.56%)	2 (2.22%)	0.631*
*C. difficile *	14 (25.92%)	12 (13.33%)	0.048
Diarrheagenic* E. coli *	7 (12.96%)	9 (10.00%)	0.594
*Shigella *spp.	3 (5.56%)	1 (1.11%)	0.148*
*C. albicans *	25 (46.30%)	12 (13.33%)	0.0001

*P value calculation using Fisher's exact test.
